# Inducible VEGF Expression by Human Embryonic Stem Cell-Derived Mesenchymal Stromal Cells Reduces the Minimal Islet Mass Required to Reverse Diabetes

**DOI:** 10.1038/srep09322

**Published:** 2015-03-30

**Authors:** E. Hajizadeh-Saffar, Y. Tahamtani, N. Aghdami, K. Azadmanesh, M. Habibi-Anbouhi, Y. Heremans, N. De Leu, H. Heimberg, P. Ravassard, M. A. Shokrgozar, H. Baharvand

**Affiliations:** 1 National Cell Bank, Pasteur Institute of Iran, Tehran, Iran; 2 Department of Stem Cells and Developmental Biology at Cell Science Research Center, Royan Institute for Stem Cell Biology and Technology, ACECR, Tehran, Iran; 3 Department of Molecular Virology, Pasteur Institute of Iran, Tehran, Iran; 4 Diabetes Research Center, Vrije Universiteit Brussel, Brussels, Belgium; 5 Biotechnology and Biotherapy Laboratory, University Pierre et Marie Curie, Paris, France; 6 Department of Developmental Biology, University of Science and Culture, ACECR, Tehran, Iran

## Abstract

Islet transplantation has been hampered by loss of function due to poor revascularization. We hypothesize that co-transplantation of islets with human embryonic stem cell-derived mesenchymal stromal cells that conditionally overexpress VEGF (hESC-MSC:VEGF) may augment islet revascularization and reduce the minimal islet mass required to reverse diabetes in mice. HESC-MSCs were transduced by recombinant lentiviruses that allowed conditional (Dox-regulated) overexpression of VEGF. HESC-MSC:VEGF were characterized by tube formation assay. After co-transplantation of hESC-MSC:VEGF with murine islets in collagen-fibrin hydrogel in the omental pouch of diabetic nude mice, we measured blood glucose, body weight, glucose tolerance and serum C-peptide. As control, islets were transplanted alone or with non-transduced hESC-MSCs. Next, we compared functional parameters of 400 islets alone versus 200 islets co-transplanted with hESC-MSC:VEGF. As control, 200 islets were transplanted alone. Metabolic function of islets transplanted with hESC-MSC:VEGF significantly improved, accompanied by superior graft revascularization, compared with control groups. Transplantation of 200 islets with hESC-MSC:VEGF showed superior function over 400 islets alone. We conclude that co-transplantation of islets with VEGF-expressing hESC-MSCs allowed for at least a 50% reduction in minimal islet mass required to reverse diabetes in mice. This approach may contribute to alleviate the need for multiple donor organs per patient.

Islet transplantation is a promising therapy for type I diabetes, a global health concern with an annually increasing worldwide incidence of 3%^1^. Despite significant improvements by the Edmonton protocol^2^, graft function progressively decreases to result in only 44% insulin independence after three years^3^. An important reason for reduced graft function is the loss of functional islets during the first two weeks post-transplantation^4^. Islets depend on vascularization as they contain a dense network of blood vessels lined by fenestrated endothelial cells as well as an intra-islet portal system and an increased oxygen pressure compared to surrounding tissue^4^,^5^. The procedure of islet isolation destroys intra-islet vasculature, requiring 10–14 days after transplantation to rebuild. In addition, this revascularization is incomplete compared to native islets in the pancreas^6^. Delayed and incomplete revascularization is one of the major impediments leading to functional engraftment of only a small fraction of transplanted islets^7^. Correlation between islet vascularization, normal glucose homeostasis and long-term islet function is obvious^8^,^9^. Thus, more robust and rapid vascularization can improve early islet survival and function.

Several studies have demonstrated beneficial effects of mesenchymal stromal cells (MSCs) co-transplantation on islet grafts^10^,^11^,^12^ via various mechanisms such as immunomodulation^13^, maintenance of islet organization^11^,^14^ and enhancement of revascularization^10^,^15^,^16^ through secretion of vascular endothelial growth factor (VEGF), hepatocyte growth factor, platelet-derived growth factor^16^,^17^ and matrix metalloproteases^18^. Furthermore, MSCs recruit and activate endogenous progenitors to promote repair of injured tissue^19^. Human embryonic stem cell-derived MSCs (hESC-MSCs), as an unlimited source of MSCs, can circumvent practical challenges that occur with the use of other routine sources of MSCs, including lack of potency, inconsistency, necessity for pathogen screening with each donor, and impaired proliferation and secretion of MSCs from diseased and old donors^20^,^21^.

Previous studies have shown a critical role for VEGF in initiating islet revascularization and increasing vascular permeability^22^,^23^ in addition to maintenance of normal islet vascular function^24^. However, excess levels of VEGF exert deleterious effects on islet function^25^,^26^. In this study, hESC-MSCs, transduced to conditionally express VEGF (called hESC-MSC:VEGF), were co-transplanted with islets in a collagen-fibrin hydrogel in the omental pouch of diabetic nude mice in order to augment islet revascularization, thereby potentially reducing the amount of islets required to reverse diabetes in mice.

## Results

### Inducible expression of VEGF through hESC-MSCs

MSCs spontaneously differentiated from hESCs in Matrigel with bFGF, were transduced with recombinant lentiviruses that allowed conditional, rtTA-mediated expression of TetO-controlled VEGF (Le-rtTA and Le-TetO-VEGF).

**Figure 1:**
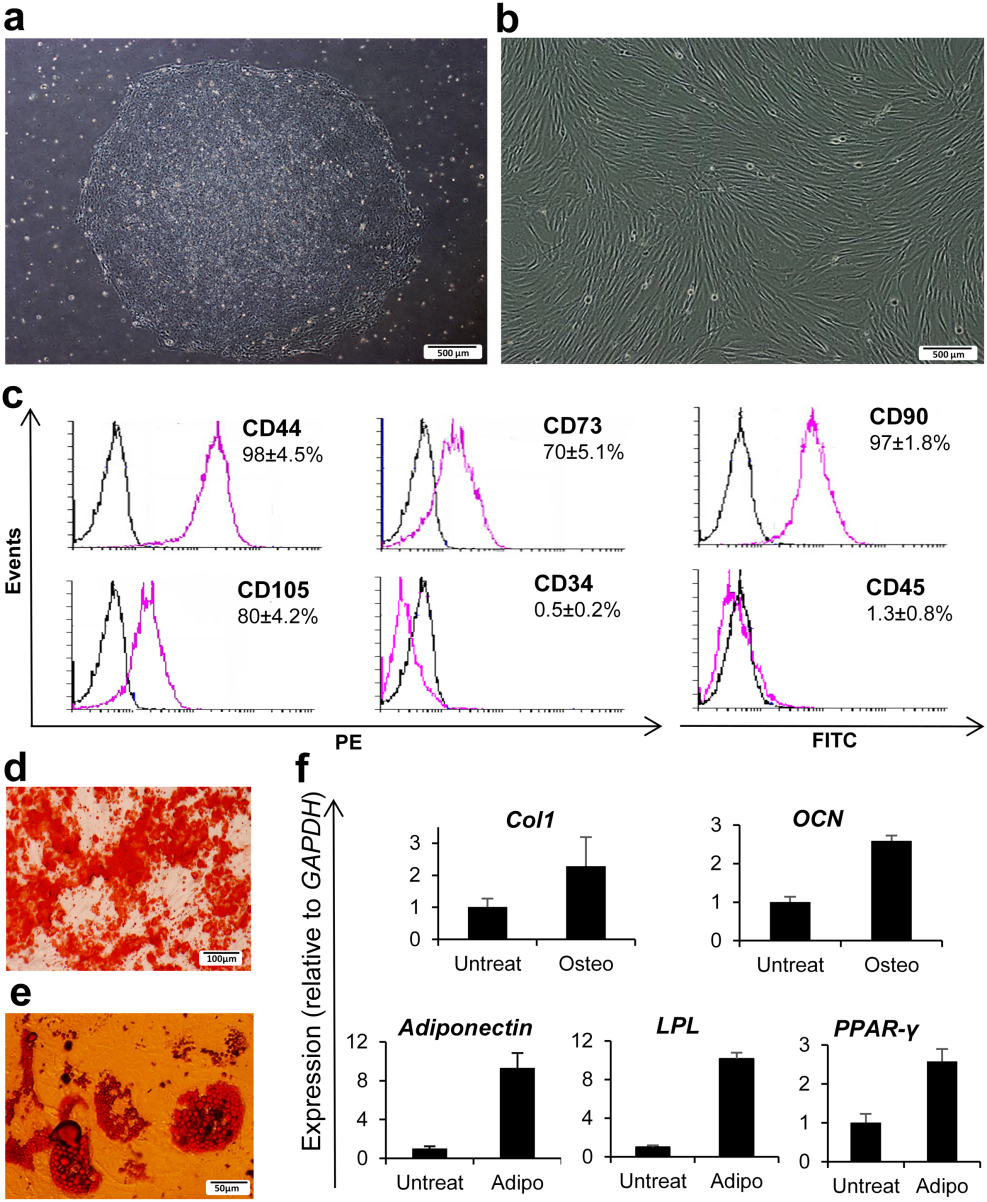
Derivation and characterization of hESC-MSCs. (a) hESC colony. (b) hESC-MSCs at passage 3. (c) Immunophenotyping of hESC-MSCs for hematopoietic and mesenchymal markers. (d) Osteogenesis of hESC-MSCs (alizarin red staining). (e) Adipogenesis of hESC-MSCs (oil red-O staining). (f) Q-RT PCR for osteocyte and adipocyte markers. FITC: fluorescein isothiocyanate; PE: phycoerythrin, *Col1: collagen type I, OCN: osteocalcin, LPL: lipoprotein lipase, PPAR-Gamma: peroxisome proliferator-activated receptor gamma, GAPDH: glyceraldehyde 3-phosphate dehydrogenase.*

Cultured hESC-MSCs showed MSC characteristics such as plastic adherence and spindle-shaped morphology, indicative for epithelial to mesenchymal transition (Figure 1b). Hematopoietic surface markers CD34 (0.5 ± 0.2%) and CD45 (1.3 ± 0.8%) were nearly absent while mesenchymal surface markers CD44 (98 ± 4.5%), CD90 (97 ± 1.8%), CD73 (70 ± 5.1%) and CD105 (80 ± 4.2%) were expressed by the majority of hESC-MSCs (Figure 1c).

Lineage differentiation of hESC-MSCs demonstrated adipogenic potential, indicated by oil-red staining of lipid droplets in the cytoplasm, and osteogenic capacity, illustrated by alizarin red staining of the extracellular calcium deposits (Figures. 1d,e), further confirmed by increased expression of adipocyte- and osteocyte-related genes as compared with untreated cells (Figure 1f). hESC-MSCs were not tumorigenic as proven by the absence of teratoma or tumor formation when compared to hESCs (Supplementary Figure S1 online).

Because of the low transduction efficiency under polybrene-free conditions (Supplementary Figure S2-a online)^27^, we optimized the polybrene concentration (range 0-10 μg/ml) according to its effect on viability and proliferation capacity of hESC-MSCs (Supplementary Figures S2-b, c online) and selected 8 μg/ml.

**Figure 2:**
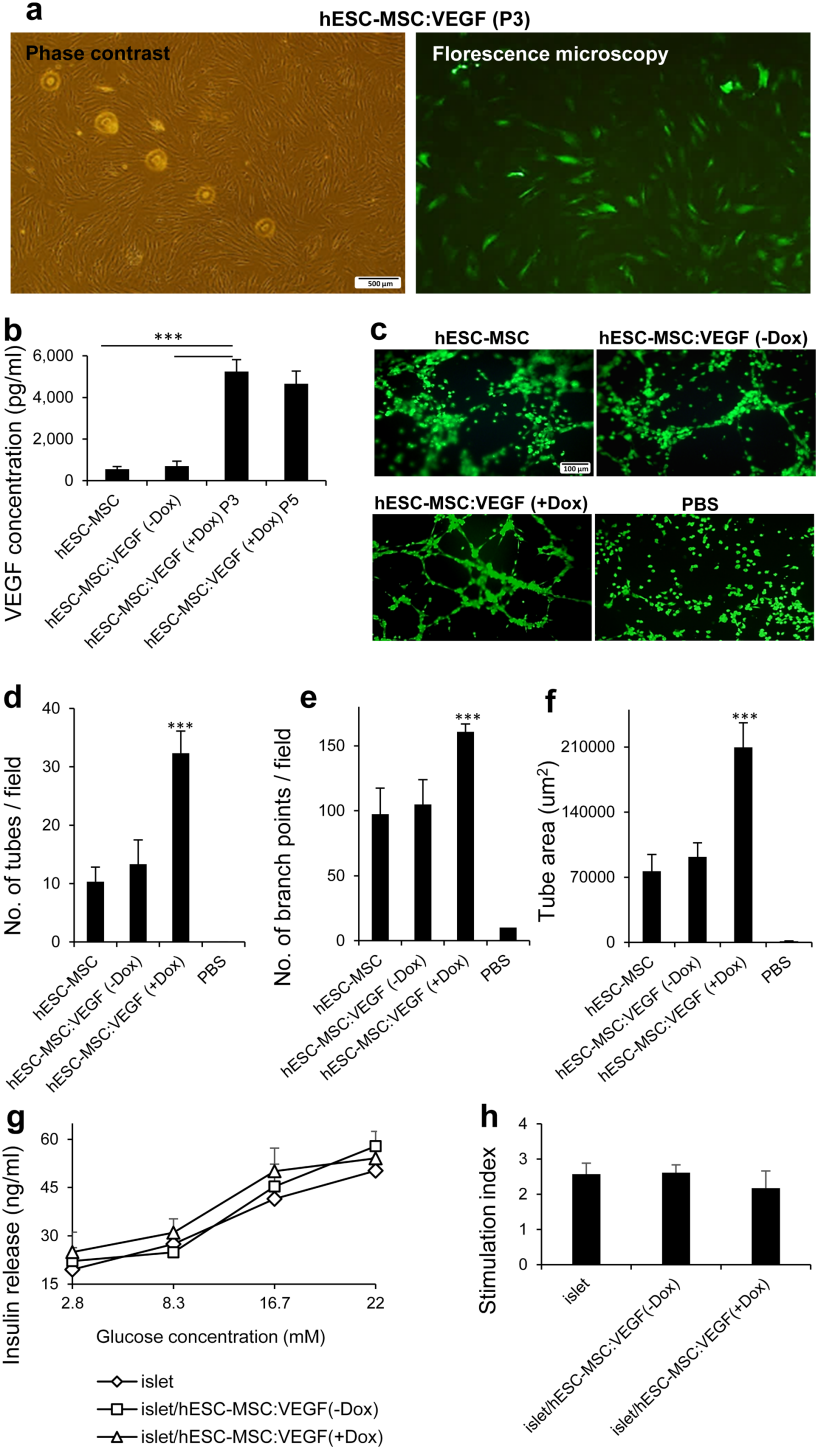
Lentiviral transduction of hESC-MSCs for inducible expression of VEGF. (a) Phase contrast (left) and fluorescent (right) images of double transduced hESC-MSCs at passage 3. (b) VEGF ELISA for transduced hESC-MSCs (hESC-MSC:VEGF) in +Dox (passages 3 and 5) or -Dox. (c) Tube formation assay (HUVECs stained by Calcein AM) and quantification by Image J, calculating the numbers of tubes (d) branch points (e) and tube area per field (f); five fields per sample, n = 3. (g) Glucose stimulated insulin secretion in 2.8, 8.3, 16.7 and 22 mM glucose and (h) Stimulation index (ratio of highly stimulated over basal insulin secretion) of islets co-cultured with hESC-MSC:VEGF. Values represent mean ± SD, ***p < 0.005. Dox: Doxycycline.

HESC-MSCs were transduced with rtTA- and TetO-VEGF-expressing lentiviruses at three different multiplicities of infection (MOIs; 5, 25 and 50). Since both lentiviruses constitutively expressed GFP, we determined the percentage of transduced cells by flow cytometry. The results showed significantly higher transduction rate with Le-rtTA at all MOIs (82 ± 9.7% at MOI 50) compared to Le-TetO-VEGF lentivirus (38 ± 9% at MOI 50; Supplementary Figure S2-d online). Therefore, VEGF-transduced hESC-MSCs were sorted (Supplementary Figure S2-e online) and subsequently transduced with Le-rtTA. The resulting cells showed normal morphology and expansion potential and were used for co-transplantation experiments when they reached passage three (Figure 2a).

VEGF secreted by hESC-MSC:VEGF at passage 3 (transplantation stage) was measured by ELISA of the medium. VEGF concentration following Dox induction (5245 ± 413 pg/ml) was seven-fold higher than without Dox induction (711 ± 98 pg/ml). VEGF secretion was not significantly altered at passage 5 (4664 ± 389 pg/ml, n = 4, p < 0.005; Figure 2b).

To functionally evaluate inducible secretion of VEGF by hESC-MSC:VEGF, a tube formation assay was performed (Figure 2c). The average number of tubes per field was significantly (n = 3; p < 0.001) higher from hESC-MSC:VEGF +Dox (32.3 ± 3.7) versus –Dox (13.4 ± 4.1). Similarly, the number of branch points per field in +Dox was higher (160 ± 19.1) versus -Dox (104 ± 18.2; n = 3; p < 0.005) as well as the average tube area for +Dox (209,709 ± 2629 μm^2^ in +Dox versus 102,908 ± 9345 μm^2^ in –Dox; n = 3; p < 0.005; Figures 2d–f).

Furthermore, in vitro glucose stimulated insulin secretion (GSIS) test was performed to examine possible effects of inducible expression of VEGF on islet secretory function. High glucose (22 mmol/l) stimulated insulin secretion in isolated islets (50.21 ± 4.02 ng/ml) was not significantly different from related result from islets co-cultured with hESC-MSC:VEGF in absence (57.87 ± 4.63 ng/ml) or presence (54.09 ± 2.76 ng/ml) of Dox induction (p > 0.1; Figure 2g). Related stimulation index was not statistically different between test and control groups (p > 0.1; Figure 2h).

### Co-transplantation of hESC-MSC:VEGF improves the function of transplanted islets

**Figure 3:**
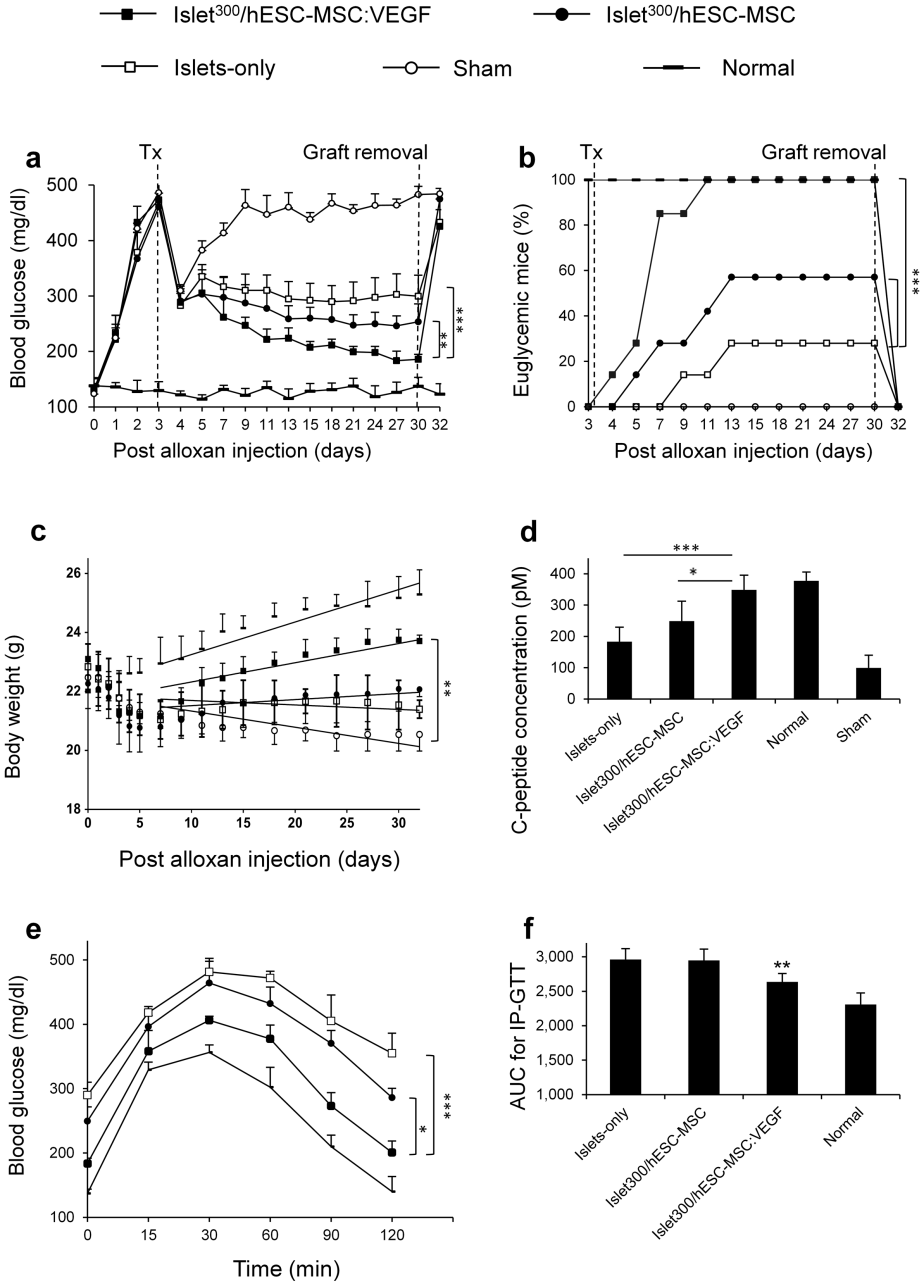
Improvement of islet function by co-transplantation of hESC-MSC:VEGF. (a) Blood glucose (mg/dl), (b) percentage of euglycemic mice and (c) body weight (g) of mice from days 0 to 32 post-alloxan injection (PAI). (d) Serum C-peptide (pM), (e) IP-GTT curve and (f) Area under curve (AUC) of IP-GTT at 30^th^ day PAI. Values represent mean ± SD, n = 7/group. * p < 0.05, **p < 0.01, ***p < 0.005. Tx: Transplantation.

For induction of diabetes, nude mice were treated with 90 mg/kg alloxan, showed diabetes induction rate of 100%, without any alloxan-induced mortality. The quality of isolated murine islets for transplantation was determined by analysis of intact morphology of the peri-islet capsule and a sample of islets was stained by dithizone (DTZ) to evaluate purity (Supplementary Figures. S4–c, d online). Average blood glucose concentration was lower in islet^300^/hESC-MSC:VEGF mice versus islets-only (p < 0.001, N = 7) or islet^300^/hESC-MSC (p < 0.01, N = 7) mice, at different time points (Figure 3a). Post-operative transient hypoglycemia due to fasting and surgery was observed in all groups. Four weeks after transplantation, 100% of mice in the islet^300^/hESC-MSC:VEGF group restored euglycemia compared to 57% in islet^300^/hESC-MSC and 28% in islets-only mice (both p < 0.001, N = 7, Figure 3b). The time to achieve euglycemia was 5 ± 2 days after transplantation for islet^300^/hESC-MSC:VEGF mice compared to 8 ± 2.5 days for islet^300^/hESC-MSC and 11 ± 3 for islets-only mice.

Regardless of initial weight loss attributed to diabetes induction and surgery until post-alloxan injection (PAI) 7, a trends towards weight gain was observed in the islet^300^/hESC-MSC:VEGF (28.6 ± 0.8 mg/day) mice compared to sham (p < 0.01, N = 7). There was no significant difference observed with the normal group (31.1 ± 0.6 mg/day; p > 0.1, N = 7; Figure 3c). Serum mouse C-peptide levels were measured, four weeks after transplantation, as an index of islet secretory activity. Very low C-peptide levels (95 ± 29 pM) were observed in non-transplanted diabetic mice, indicative for near total ablation of beta cell activity. Serum C-peptide was higher in the islet^300^/hESC-MSC:VEGF mice (349.8 ± 51.2 pM) compared to the islet^300^/hESC-MSC (249.7 ± 69.1 pM; p < 0.05; N = 7) and islets-only mice (183.2 ± 47.6 pM; p < 0.001; N = 7), but similar to normal mice (377.6 ± 41 pM; p > 0.5; N = 7; Figure 3d).

In order to examine glucose responsiveness of the islet graft, intraperitoneal glucose tolerance test (IP-GTT) was performed four weeks after transplantation. Islet^300^/hESC-MSC:VEGF mice cleared glucose at the same rate as normal mice, whereas mice in the islet^300^/hESC-MSC and islets-only mice responded more slowly and did not return to normoglycemia (Figure 3e). To validate the results of the IP-GTT, area under the curve (AUC) was calculated and found to be significantly smaller for islet^300^/hESC-MSC:VEGF versus islet^300^/hESC-MSC and islets-only mice (p < 0.01; N = 7; Figure 3f). These results indicate a significant improvement in islet function, secretory activity and glucose responsiveness after transplantation with hESC-MSC:VEGF +Dox when compared with islets transplanted in the presence or absence of hESC-MSCs.

### Co-transplantation of hESC-MSC:VEGF with islets augments graft revascularization

**Figure 4:**
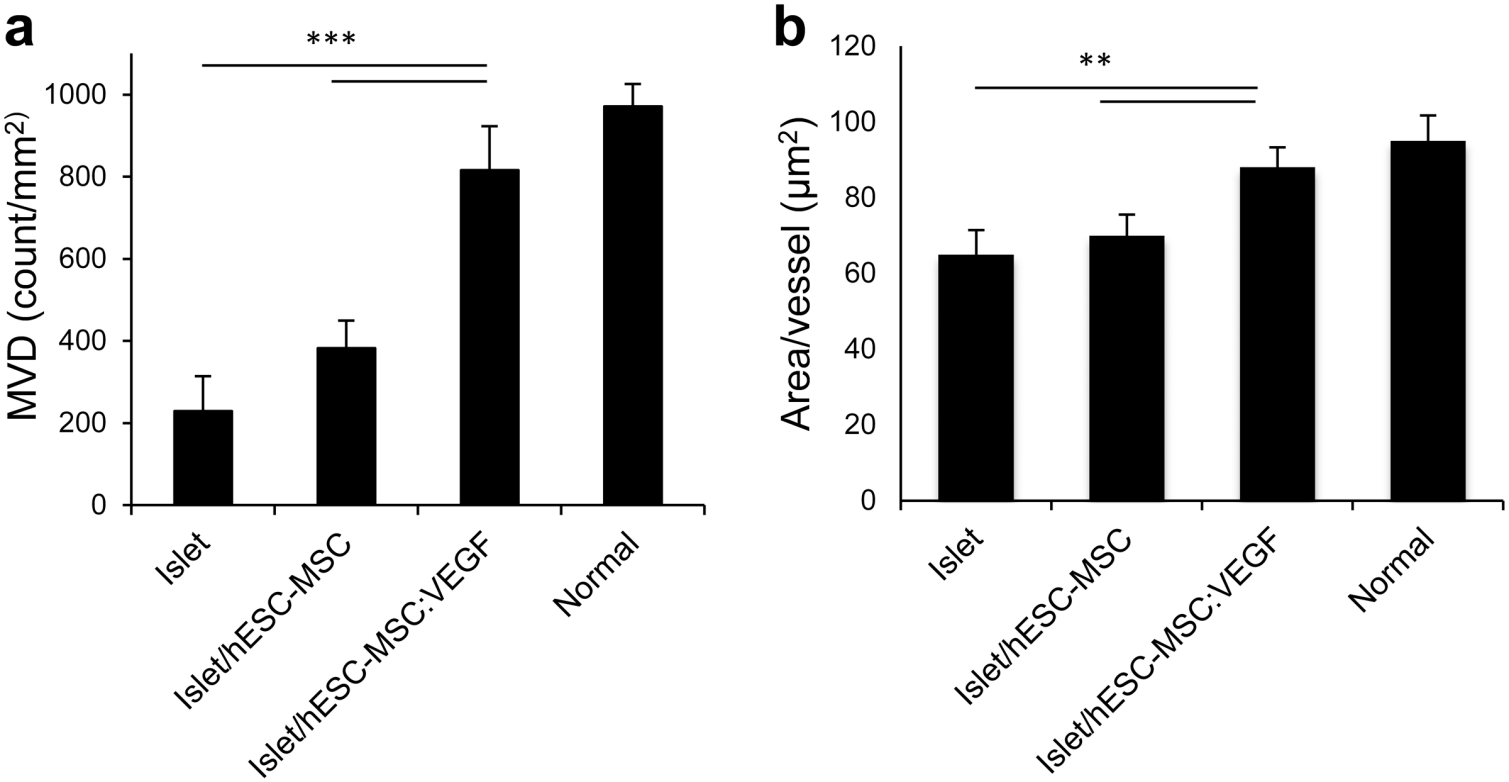
Augmentation of graft revascularization by co-transplantation of hESC-MSC:VEGF. (a) Microvessel density (MVD; count/mm^2^) as average number of microvessels in five fields of five tissue sections. (b) Area per vessel (μm^2^) calculated by dividing total vessel area (measured by Image J) to the number of vessels per field. Values represent mean ± SD, n = 7/group. * p < 0.05, **p < 0.01, ***p < 0.005.

Enhanced graft revascularization was observed in islet^300^/hESC-MSC:VEGF compared to islet^300^/hESC-MSC and islets-only mice, at four weeks after transplantation. Functional vessels were labeled by intravenous injection of FITC-conjugated lectin (Supplementary Figure S6-a online). Furthermore, graft sections were co-stained with insulin and platelet endothelial cell adhesion molecule (PECAM-1) antibodies (Supplementary Figure S6-b online). In grafts of islet^300^/hESC-MSC:VEGF mice 816.4 ± 47 vessels were detected per mm^2^, markedly higher than islet^300^/hESC-MSC (381.8 ± 56/mm^2^) and islets-only (229.1 ± 51/mm^2^) mice (both p < 0.001; Figure 4a). Another revascularization index, area per vessel, was significantly higher for grafts in islet^300^/hESC-MSC:VEGF mice (88.1 ± 5.2 μm^2^), than in islet^300^/hESC-MSC (70 ± 5.4 μm^2^) and islets-only (65 ± 6.1 μm^2^) mice (n = 7, p < 0.01, Figure 4b). Notably, both indices measured in islet^300^/hESC-MSC:VEGF mice showed no significant differences compared to the normal mice (p > 0.1). These results indicate not only the presence of more functional vessels in the islet^300^/hESC-MSC:VEGF grafts, but also the presence of vessels that are larger and more similar to endogenous islets of normal group as compared to the vasculature in the grafts of islet^300^/hESC-MSC or islets-only mice.

### Inducible expression of VEGF through hESC-MSC:VEGF causes at least a 50% reduction in the minimal islet mass required to reverse diabetes

In the second phase of the study, we hypothesized that the beneficial effect of inducible expression of VEGF through hESC-MSC:VEGF on islet revascularization could lead to a reduction in minimal islet mass required to reverse diabetes in mice. In this regard, islet function was compared between mice transplanted with 400 islet equivalent (IEQ) versus 200 IEQ + hESC-MSC:VEGF.

**Figure 5:**
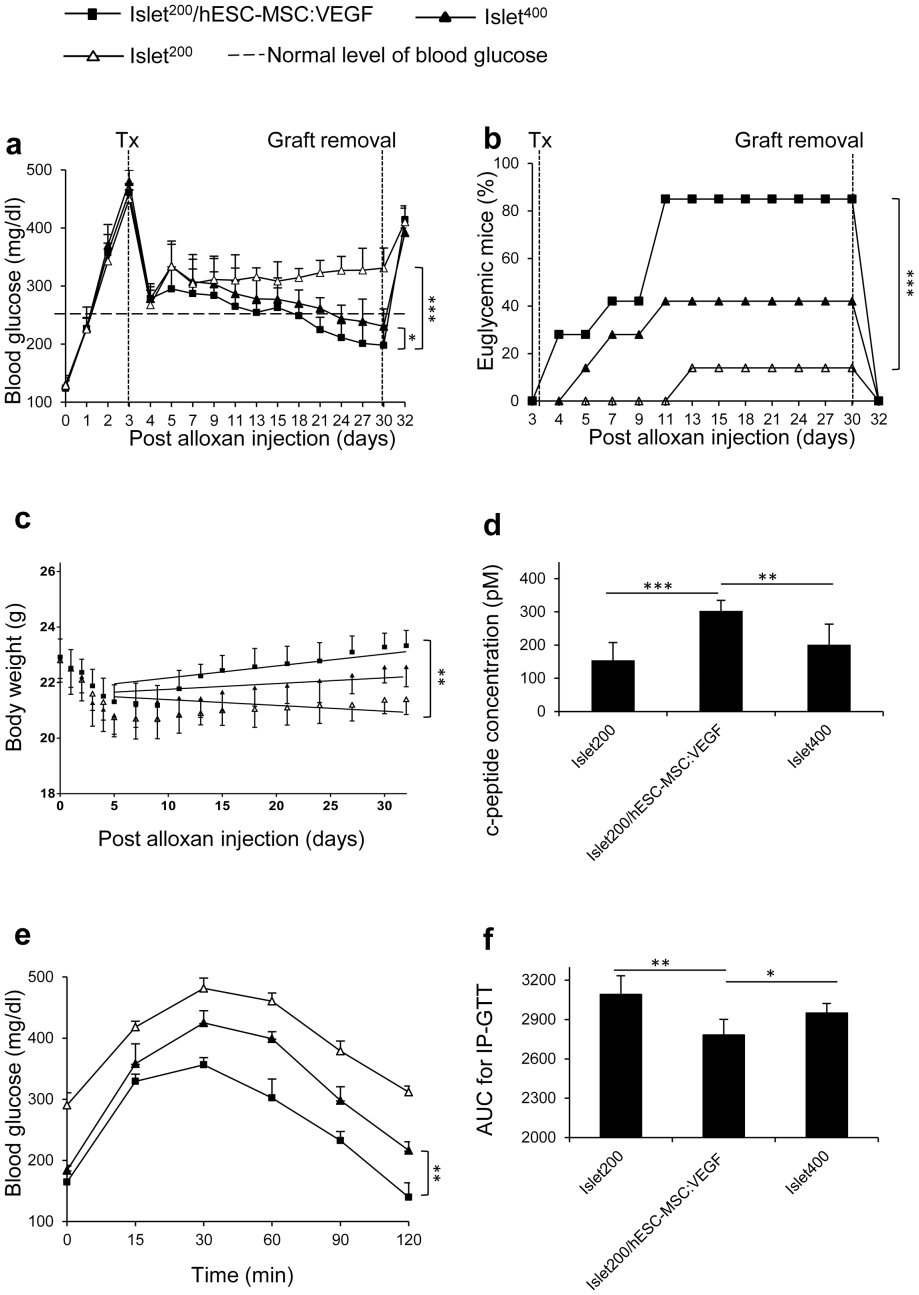
Reduction in minimal islet mass required to reverse diabetes by co-transplantation of hESC-MSC:VEGF. (a) Blood glucose (mg/dl), (b) percentage of euglycemic mice and (c) body weight in grams (g) of mice from days 0 to 32 PAI. (d) Serum C-peptide (pM), (e) IP-GTT curve and (f) AUC of IP-GTT of mice at 30^th^ day PAI. Values represent mean ± SD, n = 7/group. * p < 0.05, **p < 0.01, ***p < 0.005. Tx: Transplantation.

Islet function was superior in the islet^200^/hESC-MSC:VEGF group compared to the islet^400^ group. Blood glucose concentrations of mice that received islet^200^/hESC-MSC:VEGF were significantly lower than those of the islet^400^ group (p < 0.05; N = 7) at different time points (Figure 5a). Similarly, the percentage of euglycemic mice was higher in islet^200^/hESC-MSC:VEGF (85%) compared to islet^400^ group (57%)(p < 0.001; N = 7), at four weeks after transplantation (Figure 5b). The mild trend toward weight gain in islet^200^/hESC-MSC:VEGF group (21.5 ± 0.4 mg/day) until PAI 7 was significantly higher than in islet^200^ group (p < 0.01; N = 7), but was not different from islet^400^ group (p > 0.5; N = 7; Figure 5c).

The serum mouse C-peptide level was higher in islet^200^/hESC-MSC:VEGF group (302.8 ± 31 pM) compared to islet^400^ group (201.7 ± 60.5 pM; p < 0.01; N = 7; Figure 5d). The same pattern was observed for IP-GTT: mice in islet^400^ group responded more slowly than islet^200^/hESC-MSC:VEGF mice. The related AUC for islet^200^/hESC-MSC:VEGF group was smaller than the islet^400^ group (p < 0.05; N = 7; Figures. 5e, and f). These data indicate that 200 IEQ transplanted with hESC-MSC:VEGF show superior function over 400 IEQ-only. This co-transplantation thus caused at least a 50% reduction in minimal islet mass required to reverse diabetes in mice.

## Discussion

Delayed and incomplete graft revascularization impedes the clinical outcomes of islet transplantation, and results in the need for multiple donor organs per patient^7^,^28^. Here, we co-transplanted islets with VEGF expressing hESC-MSCs in a collagen-fibrin hydrogel in the omental pouch of diabetic nude mice, to evaluate their effect on islet revascularization and function.

Advantages of MSCs co-transplantation on islet grafts^10^,^11^,^12^,^16^ include immunomodulation^13^,^29^ and paracrine secretion of angiogenic factors^16^. The first possibility is unlikely to occur in the current study due to the use of nude diabetic mice. Although we observed a number of beneficial effects like reduction of blood glucose in islet/hESC-MSC mice, our observations do not support the idea that co-transplantation of islets with hESC-MSCs is sufficient for complete graft revascularization and reversal of hyperglycemia in diabetic nude mice. Islets themselves secrete some angiogenic factors^30^, which likely explain the lesser revascularization that we have seen in islets-only group, but it may cause delayed revascularization under hyperglycemia^31^, a common condition of diabetic patients, this could justify the need to increased dose of angiogenic factors around transplanted islets. The angiogenic effects of VEGF in islets have been previously shown^23^,^30^, excessive levels of VEGF have negative effects including impaired glucose stimulated insulin secretion from islets^25^,^26^. Therefore, the strategy of local^32^ and inducible^33^ expression of VEGF has been suggested to eliminate harmful effects of systemic administration^34^ or sustained high levels of VEGF during islet transplantation studies. In the current study, we have established an inducible tet-ON system that delivers VEGF locally from hESC-MSC:VEGF solely during the first two weeks after islet transplantation, a period considered crucial for complete formation of new blood vessels^35^. Meanwhile the use of a pTight promoter in the construction of the VEGF containing lentivirus precludes background expression of VEGF in the absence of Dox. Furthermore, in vitro and in vivo obtained results on GSIS potential of islets, co-cultured or co-transplanted with hESC-MSC:VEGF, proved that released VEGF from mentioned cells does not exceed its permissible dose to negatively affect GSIS. Regarding some concerns surrounding the use of lentiviral gene delivery to transplanted cells in clinical setting, there are several released results from phase I/II clinical trials indicates safety of this therapy in the follow up period^36^,^37^,^38^.

The omental pouch was chosen as the transplantation site because of its rich, dynamic blood supply and the presence of pro-angiogenic cytokines^39^. In man, omentum is a large, non-vital easily accessible organ which is considered as promising extra-hepatic site for islet transplantation^8^. Evidence has shown that (a combination of) specific extracellular matrix components in an islet graft improve islet function^40^. Among extracellular components, fibrin is a biodegradable, clinically acceptable, ideal biomaterial for vascularization^41^,^42^. We have chosen combinatory hydrogel which has superior functional and mechanical properties to individual hydrogel^43^,^44^,^45^, mainly because it consists of fibrin with natural cell binding sites that promote vascularization^46^ and type I collagen that lends physical support as a structural protein, while its other characteristics are beneficial for islet graft function^47^. In addition, a combinatory hydrogel consists of a distinct collagen-fibrin ratio (2:3), which acts as potent as pure fibrin for endothelial cell network formation^45^,^48^.

We have demonstrated inducible secretion of bioactive VEGF from hESC-MSC:VEGF +Dox grafts that improves vascularization and function of the grafted islets. Our results indicate that not only more microvessels grew in grafts of the islet^300^/hESC-MSC:VEGF mice, but also these functional vessels were larger in size and similar to islets of normal mice.

In the second phase of the study, we observed at least a 50% reduction in the minimal mass of islets required to reverse diabetes in mice. In this regard, islet function parameters were superior in the islet(200)/hESC-MSC:VEGF group compared with the islet^400^ group. As the function of transplanted islets is dependent on their optimum engraftment and vascularization, this result is likely mediated through enhancement of revascularization, observed in the first phase of the study, and probably due to other paracrine effects of hESC-MSC:VEGF on transplanted islets. While the focus of this study is on the angiogenic effects of inducible VEGF expression, there are some reports which show the time- and dose-dependent effects of VEGF induction on proliferation and regeneration of pancreatic islets^33^,^49^. Clinical islet transplantation should proceed toward the use of single instead of multiple donor organs^28^. Therefore any improvements in transplanted islet function can be clinically important by reducing the amount of islets required, increasing the insulin independence following islet transplantation from single pancreas donor, and decreasing procedural complications attributed to multiple islet infusions.

In summary, we showed improved function of islets when co-transplanted with hESC-MSC:VEGF +Dox and superior revascularization compared with islet^300^/hESC-MSC and islets-only mice. The results of the current study show that such co-transplantation could reduce the minimal islet mass required to reverse diabetes in mice by half. However, a number of safety concerns exist in the use of hESCs and its derivatives in clinical trials. These results should be further investigated in the context of allogeneic islet transplantation with the intent to obtain proper glyco-metabolic control with islets from a single organ donor.

## Methods

### Derivation and culture of hESC-MSCs

The hESC line, Royan H6 (Royan Stem Cell Bank, Tehran, Iran) at passage 50–55 was used in this study. RH6 colonies were cultured on Matrigel-coated dishes (Sigma-Aldrich, St. Louis, MO, USA) in the presence of 100 ng/ml basic fibroblast growth factor (bFGF; Sigma-Aldrich) as previously described^50^. The hESC medium was changed every day until day four after which the medium changes were performed every three days to induce spontaneous differentiation of hESCs toward MSCs^51^. After a brief exposure of colonies to a collagenase IV/dispase mixture solution (1:2, v/v; both from Gibco, Gaithersburg, MD, USA), the central areas of the colonies were removed by scraping with a bent Pasteur pippete and suctioning. The remaining edge of colonies that consisted of spindle-shaped cells were treated with 0.05% trypsin/EDTA (Invitrogen, Carlsbad, CA, USA) and transferred to MSC medium that contained Alpha Modified Eagle's Medium (αMEM) supplemented with 15% FBS, 1 mM nonessential amino acids, 2 mM l-glutamine, 1% penicillin/streptomycin (all from Invitrogen) and 0.1 mM B-mercaptoethanol (Sigma-Aldrich). MSC medium was changed every three days.

### Surface marker analysis for characterization of hESC-MSCs

For immunophenotyping of hESC-MSCs, cells were dissociated in 0.05% trypsin-EDTA and washed in PBS (Gibco) supplemented with 1% heat-inactivated FBS and 2 mM EDTA (Merck, Darmstadt, Germany). A total of 3–5 × 10^5^ cells per sample were incubated with MSC surface marker primary antibodies for two hours at 4°C and with the secondary antibodies for 30 minutes at 4°C (Supplementary Table S1 online). Control staining with appropriate isotype-matched monoclonal antibodies was included in all experiments. Flow cytometric experiments were performed in triplicate with a BDFACS Calibur Flow Cytometer (BD Biosciences, Franklin Lakes, NJ, USA). Acquired data were analyzed by WinMDI 12.9 software (freeware from Joe Trotter, The Scripps Research Institute, La Jolla, CA, USA).

### Lineage differentiation and Q-RT-PCR for characterization of hESC-MSCs

For lineage differentiation of hESC-MSCs, 1 × 10^4^ cells were seeded per well of six-well plates (TPP, Trasadingen, Switzerland) and treated with osteogenic or adipogenic medium for 21 days with media changes every three days. For osteogenic induction, 1 μM dexamethasone, 0.5 μM ascorbic acid, and 10 mM b-glycerol phosphate (all from Sigma-Aldrich) and for adipogenic induction 50 μg/ml indomethacin (Sigma-Aldrich), 50 μg/ml ascorbic acid, and 100 nM dexamethasone were added to MSC medium. Osteogenesis was assessed by alizarin red (Sigma-Aldrich) and adipogenesis by oil red-O (Sigma-Aldrich) staining.

For Q-RT-PCR analysis, total RNA of the differentiated osteocytes and adipocytes was isolated by RNeasy Kit (Qiagen, Germantown, MD, USA) and used for reverse transcription reaction with the RevertAid First Strand cDNA Synthesis Kit (Fermentas, Vilnius, Lithuania), according to the manufacturer's instructions. Three independent experiments were performed and reactions were run in duplicate by the use of a Power SYBR Green Master Mix (Applied Biosystems, Foster City, USA). Obtained results were analyzed with the 7500 real-time PCR system (Applied Biosystems) and expression values of human collagen type I (Col1), osteocalcin (OCN), adiponectin, lipoprotein lipase (LPL) and peroxisome proliferator-activated receptor gamma (PPAR-gamma) were normalized to the average expression of the housekeeping gene glyceraldehyde 3-phosphate dehydrogenase (GAPDH) by the comparative CT method. Sequences of related primers are shown in Supplementary Table S2 online.

### Tumorigenicity test for characterization of hESC-MSCs

In order to evaluate tumorigenicity of hESC-MSCs, 2 × 10^6^ of either RH6 hESCs or hESC-MSCs were dissociated, mixed with 30 μL Matrigel (diluted 1:1 in αMEM) and subsequently transplanted under the testis capsule of nude mice. After 15 weeks post-transplantation, hematoxylin and eosin (H&E) staining was performed on 10% formaldehyde-fixed, paraffin-embedded testes samples and the results compared with the hESCs group.

### Transduction of hESC-MSCs with rtTA and VEGF lentiviruses

In order to induce conditional expression of VEGF by hESC-MSCs, a dual lentiviral system was used (Le-rtTA and Le-TetO-VEGF-A, both also expressing GFP for quantitation of transduction efficiency). VEGF-A expression in the Le-TetO-VEGF-A construct is under control of the pTight promoter, preventing leaky expressing of VEGF-A (Beta Cell Therapy Virus Core Facility, Biotechnology and Biotherapy Group, CNRS, Hospital de la Pitié Salpêtrière, Paris, France). To optimize transduction conditions, hESC-MSCs were exposed to different doses of polybrene (Sigma-Aldrich) that ranged from 0–10 μg/ml for 16 hours. Viability of cells was assessed by flow cytometric measurement of fluorescence after staining with propidium iodide (PI; Sigma-Aldrich) and their proliferation capacity was analyzed by comparing the results of the MTS assay on day 8 to day 1. For the MTS assay, 10^4^ cells were seeded per well of a 96-well plate and incubated for 2 hours in the presence of 20 μl MTS reagent (Thermo Scientific, Waltham, MA, USA) at 37°C. Optical density was read at 490 nm by MultiSkan ELISA reader (Thermo Scientific).

For lentiviral transduction, 1 × 10^5^ hESC-MSCs were seeded per well of six-well plates. In the presence of 8 μg/ml polybrene, each lentivirus was added to one ml MSC medium per well at different MOIs. Cells were incubated at 37°C for 16 hours after which transduction medium was replaced with fresh MSC medium. Mock transduction was performed under the same conditions but without the viruses. At 72 hours post-transduction, the cells were analyzed by flow cytometry to compare transduction efficiency. VEGF- transduced cells were sorted by FACS Aria II (BD Biosciences) according to GFP gating and the collected GFP+ cells were subsequently transduced with rtTA containing lentivirus.

### Quantitative and functional assays for inducible expression of VEGF

Double transduced hESC-MSCs (hESC-MSC:VEGF) or non-transduced cells were seeded at a density of 5 × 10^4^ per well of 12-well plates. VEGF expression was induced by addition of 1 mg/ml doxycycline (Dox; Clontech, Mountain View, CA, USA) to the culture medium. Tet system approved FBS (Clontech) was used to avoid background expression of VEGF. The cells were incubated at 37°C for 16 hours, then VEGF in the supernatant was measured using a mouse VEGF ELISA kit (Invitrogen). All experiments were performed in triplicate and samples were diluted 1:20 to fit a standard curve.

For functional study of inducible expression of VEGF from hESC-MSC:VEGF, tube formation assay was done using ECMatrix™ according to manufacturer's instructions (Chemicon International, MA, USA). Briefly, a 96-well plate was coated with ECMatrix during one hour at 37°C. Then, HUVECs (isolated manually according to established protocol^52^) were resuspended in conditioned medium of hESC-MSC:VEGF cultured during 16 hours with or without Dox and seeded on ECMatrix at 5 × 10^3^ cells per well. PBS was used as negative control. After six hours incubation at 37°C, cells were stained by fluorescent viable dye, Calcein AM (BD Biosciences) according to manufacturer's instructions. Tube formation in each well was examined with a fluorescence microscope (Olympus BX51, Olympus, Center Valley, PA, USA) and quantification was done using image analysis software (ImageJ; National Institutes of Health, Bethesda, MD, USA), by calculation of the number of tubes per field, number of branch points per field and tube area per field in at least five images from different areas of each sample in three independent experiments.

To evaluate possible effects of inducible expression of VEGF on islet secretory function, in vitro GSIS was measured under static condition. Freshly isolated islets were co-cultured with hESC-MSC:VEGF in presence or absence of Dox induction, with the distinct ratio similar to in vivo transplant (10 IEQ and 3 × 10^3^ cells per well) for 24 hours. A group of islets without cell co-culture was used as control and five wells per condition were run. Each batch of islets was preincubated for 30 min in Krebs-Ringer buffer containing 10% BSA and 2.8 mmol/l glucose at 37°C and subsequently incubated for one hour with 2.8, 8.3, 16.7 and 22 mmol/l glucose. Insulin was measured in the supernatant with Rat/mouse Insulin ELISA kit (Millipore). The stimulation index was calculated as the ratio of highly stimulated (22 mmol/l glucose) over basal insulin secretion (2.8 mmol/l glucose).

### Mouse pancreatic islet isolation and quality assessment

Islets were isolated from C57BL/6 mice (provided from Pasteur Institute, Tehran, Iran) using our standard procedures. In brief, five ml of 0.74 mg/ml collagenase type V (Sigma-Aldrich) in RPMI-1640 medium (Gibco) was infused through the common bile duct (Supplementary Figure S4-a online). After complete distention of the pancreas, it was dissected from the surrounding tissue (Supplementary Figure S4-b online) and further digested in collagenase solution at 37°C during 14 minutes. Then, ice-cold RPMI-1640 supplemented with 10% FBS, 2 mM l-glutamine and 1% penicillin/streptomycin was added to stop digestion. The tissue was further dissociated by shaking. After washing, undigested tissue fragments were removed by filtration over a 500 μm wire mesh and islets were purified by gradient density using Histopaque® (Sigma-Aldrich), followed by manual islet picking to ensure high purity. Islets were cultured overnight in RPMI-1640 + 10% FBS at 37°C and 5% CO2. Islet capsule integrity was assessed by microscopy, the purity was analyzed by dithizone (DTZ, Sigma-Aldrich) staining and IEQ were measured before transplantation^53^.

### Diabetes induction and transplantation of islet-containing hydrogel

Diabetes was induced in 4 hours fasted, eight-week-old male nude mice (B6NU, provided from Pasteur Institute) by a single tail vein injection of alloxan (Sigma-Aldrich) at 90 mg/kg body weight, freshly dissolved in alloxan buffer (1 mM HCl in 0.9% NaCl). Alloxan dose and route of administration was optimized in B6NU mice to obtain high diabetes induction and low mortality rate (Supplementary FigureS4 online). Diabetes was confirmed by blood glucose levels exceeding 350 mg/dl for two consecutive measurements.

Prior to transplantation, a desired number of islets with or without hESC-MSCs was resuspended in 100 μl of collagen-fibrin hydrogel. To construct the hydrogel, collagen I 15 mg/ml (extracted manually from rat tail), phosphate buffered saline (PBS) 10× (Sigma-Aldrich) and sodium hydroxide 0.4 N (Sigma-Aldrich) were mixed and incubated on ice for one minute. Then, human fibrinogen 15 mg/ml (Sigma-Aldrich) and thrombin 50 unit/ml (Sigma-Aldrich) were added with an insulin syringe (Supplementary FigureS5-b online). Islet-containing hydrogel was incubated during one hour at 37°C.

Transplantation was performed into the omental pouch of anesthetized recipient mice. Briefly, the mouse abdomen was shaved, prepped, and draped in sterile fashion. After a midline laparotomy, the greater omentum was spread out and sutured along its margin. Islet-containing hydrogel was gently transplanted into the pouch to avoid rupturing of the omental vasculature (Supplementary FigureS5-c online). The muscular layer was closed with 4-0 vicryl and the skin with silk suture.

### In vivo experiment design

In the first phase of the study, 300 IEQ and 10^5^ cells were used. Recipient mice were divided into three groups: i) islets only (islet^300^), ii) islet^300^/hESC-MSC and iii) islet^300^/hESC-MSC:VEGF. The experiments also included a sham group which underwent transplantation without receiving islets or cells and a weight-matched group that was left non-transplanted and normoglycemic, named as normal group.

In the second phase of the study, recipient mice were divided into three groups: i) mice that received 200 IEQ (islet^200^); ii) mice that received 200 IEQ plus 10^5^ cells (islet^200^/hESC-MSC:VEGF); and iii) mice that received 400 IEQ (islet^400^). Due to inducible expression of VEGF, group 3 in the first study phase and group 2 in the second study phase received Doxycycline in the drinking water (200 μg/ml), for the first two weeks after transplantation. All animal experiments were performed according to NIH Guidelines for Care and Use of Laboratory Animals (NIH Publication No. 85–23, revised 2010) and with the approval of the Royan Institute Ethics Committee.

### Analysis of transplanted islet function

Non-fasting blood glucose levels and body weight of recipients were measured until day 32 PAI. The percentage of euglycemic mice (blood glucose ≤250 mg/dl) and time to achieve euglycemia was determined. On day 30 PAI, serum C-peptide was measured using a mouse C-peptide ELISA kit (ALPCO Diagnostics, Windham, NH, USA) and an IP-GTT was performed. For IP-GTT, mice received 50% dextrose in Ringer's solution intraperitoneally at 2 g/kg body weight, after six hours fasting. Tail tip blood glucose levels were monitored at 0, 15, 30, 60 and 120 min post injection by an Accu-Check glucometer (Roche Diagnostics, Indianapolis, IN, USA) and the area under the IP-GTT curve (AUC) was measured.

The graft-bearing omentum was harvested and processed for histological analysis. Afterwards, mice were observed in order to confirm the functionality of the islet graft, as indicated by a return of blood glucose to diabetic levels.

### Analysis of transplanted islet vascularization

At four weeks after transplantation, mice were anesthetized with ketamine (80 mg/kg body weight) and xylazine (10 mg/kg body weight). 0.1 ml fluorescein isothiocyanate–conjugated tomato lectin (Lycopersicon Esculentum, 1 mg/ml; Vector Laboratories, Burlingame, CA, USA) was injected into the tail vein of mice. After five minutes, the islet-bearing omentum was dissected, fixed in 4% paraformaldehyde (Sigma-Aldrich) during 24 hours and embedded in paraffin. Serial sections with 5 μm thickness were mounted on charged SuperFrost® Plus slides (Thermo scientific, USA). After rehydration, antigen retrieval was done with retrieval solution (Dako, Denmark), permeablization was done with 0.5% triton X-100 (Merck) for 10 minutes and tissues were blocked with 10% donkey serum (made in house) in PBS for one hour at 37°C. Goat anti-mouse insulin primary antibody (Supplementary Table S1 online) was applied to the slides overnight at 4°C. Slides were incubated one hour at 37°C with secondary antibody (Supplementary Table S1 online). All washes and dilutions were performed using PBS containing 0.1% (wt/vol) BSA. Negative control slides were incubated with normal donkey serum instead of primary antibody. Nuclei were stained using 4′, 6-diamidino-2-phenylindole dihydrochloride (DAPI; Sigma-Aldrich).

To evaluate graft revascularization, two indices were measured. For microvessel density (MVD) measurement, five tissue sections at different graft depths, each at 150 μm interval were evaluated. The average number of microvessels (MVD) were counted at a magnification of 40× in five fields of each tissue section for three independent samples. Area per vessel was calculated by dividing the total vessel area (measured by Image J) by the number of vessels per field. This indicated the vessel size in each transplant group.

### Statistical analysis

All experimental values were expressed as mean ± standard deviation (SD) and the differences of mean values were statistically evaluated by SPSS software version 16 (SPSS Inc., Chicago, IL, USA) using one-way analysis of variance (ANOVA) followed by Bonferoni's post-hoc test. The trends of changes between groups during the measured time points were compared using the general linear model. Linear regression of body weights was measured using GraphPadPrism 6 software (GraphPad Prism, SanDiego, CA, USA). P-values less than 0.05 were considered significant.

## Author Contributions

E.H.S. performed and analyzed all experiments, E.H.S. and Y.T. wrote the main manuscript text, K.A. designed the viral transduction experiments, M.H. contributed in transduced cells assays, Y.H., N.D. and H.H. read the manuscript and commented about different sections of manuscript, P.R. designed and manufactured the lentiviruses, N.A., M.S. and H.B. contributed in scientific and operational design of experiments and writing of the manuscript.

## Supplementary Material

Supplementary Information Supplementary information
