# Supplementary material for: Inducible VEGF Expression by Human Embryonic Stem Cell-Derived Mesenchymal Stromal Cells Reduces the Minimal Islet Mass Required to Reverse Diabetes
Source: Sci Rep. 2015 Mar 30;5:9322. doi: 10.1038/srep09322 (PMC4377549; doi:10.1038/srep09322)
Supplement: Supplementary Information [file srep09322-s1.pdf]

Inducible VEGF Expression by Human Embryonic Stem Cell-Derived Mesenchymal Stromal Cells Reduces the Minimal Islet Mass Required to Reverse Diabetes

E. Hajizadeh-Saffar[1,2], Y. Tahamtani[2], N. Aghdami[2], K. Azadmanesh[3], M. Habibi-Anbouhi[1], Y. Heremans[4], N. De Leu[4], H. Heimberg[4], P. Ravassard[5], M. A. Shokrgozar[1*], H. Baharvand[2, 6*]

1. National Cell Bank, Pasteur Institute of Iran, Tehran, Iran

2. Department of Stem Cells and Developmental Biology at Cell Science Research Center, Royan Institute for Stem Cell Biology and Technology, ACECR, Tehran, Iran

3. Department of Molecular Virology, Pasteur Institute of Iran, Tehran, Iran

4. Diabetes Research Center, Vrije Universiteit Brussel, Brussels, Belgium

5. Biotechnology and Biotherapy Laboratory, University Pierre et Marie Curie, Paris, France

6. Department of Developmental Biology, University of Science and Culture, ACECR, Tehran, Iran

***Corresponding authors:**

Hossein Baharvand

Department of Stem Cells and Developmental Biology at Cell Science Research Center, Royan Institute for Stem Cell Biology and Technology, ACECR, Tehran, Iran.

P.O. Box: 19395-4644

Tel: +98 21 22306485

Fax: +98 21 23562507

Email: Baharvand@Royaninstitute.org

And

Mohamad Ali Shokrgozar

National cell bank of Iran, Pasteur Institute of Iran, Farvardin St., Pasteur Sq., Tehran, Iran.

P.O. Box: 13164

Tel: +982166492595

Fax: +9821 66465132

Email: mashokrgozar@pasteur.ac.ir

SUPPLEMENTARY TABLES

Supplementary Table S1. List of antibodies used for flow cytometry and immunostaining.

Antibodies	Dilution	Cat. number	Company
**Goat anti-mouse insulin**	1:200	sc-7839	Santa Cruz
**Donkey anti-goat alexafluor 594**	1:1000	A11057	Invitrogen
**IgGI-FITC**	1:500	11-471473	eBioscience
**IgGI-PE**	1:500	11-4714	eBioscience
**Anti-human CD44-PE**	1:500	12-0441	eBioscience
**Anti-human CD73-PE**	1:500	550257	BD
**Anti-human CD105-PE**	1:500	FAB10971P	R&D Systems
**Anti-human CD34-PE**	1:500	341071	BD
**Anti-human CD90-FITC**	1:500	f7274	Dako
**Anti-human CD45-FITC**	1:500	341071	BD
**Goat anti-rabbit alexafluor 488**	1:1000	A11008	Invitrogen
**Rabbit anti-mouse PECAM-1**	1:100	SAB 1302548	Sigma-Aldrich

FITC: fluorescein isothiocyanate, PE: phycoerythrin.

Supplementary Table S2. List of primers used for Q-RT-PCR.

**Gene Symbol**	**Sequence 5`–3`, Forward**	**Sequence 5`–3`, Reverse**	**Size (bp)**	**Anneal Temp. (°C)**
Col1	GTGGTGACAAGGGTGAGACAG	CAACAGGACCAGCATCACCAG	225	62
OCN	GTG CAG AGT CCA GCA AAG GT	TCA GCC AAC TCG TCA CAG TC	175	60
Adiponectin	CCT GGT GAG AAG GGT GAG AA	CAA TCC CAC ACT GAA TGC TG	205	60
LPL	TCA ACT GGA TGG AGG AGG AG	GGG GCT TCT GCA TAC TCA AA	169	60
PPAR-γ	TCT CCA GCA TTT CTA CTC CAC	GAT GCA GGC TCC ACT TTG AT	157	61
GAPDH	GAAATCCCATCACCATCTTCC	GGCTGTTGTCATACTTCTCAT	219	61

Col1: collagen type I, OCN: osteocalcin, LPL: lipoprotein lipase, PPAR-gamma: peroxisome proliferator-activated receptor gamma, GAPDH: glyceraldehyde 3-phosphate dehydrogenase.

SUPPLEMENTARY FIGURE LEGENDS

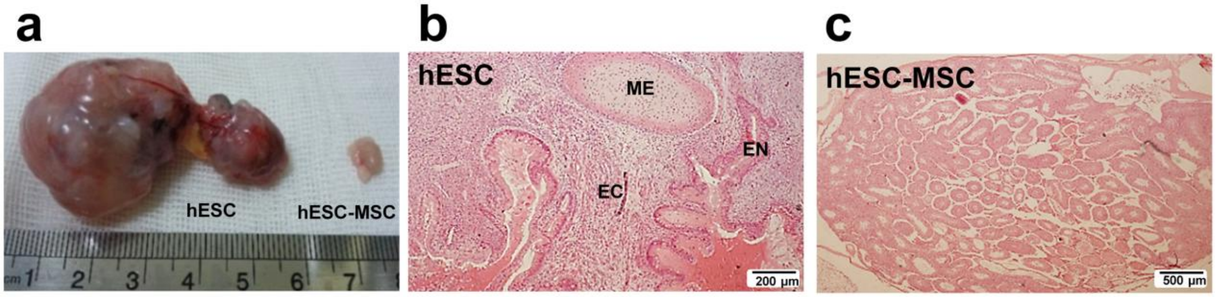

Supplementary Fig. S1. Tumorigenicity test for hESC and hESC-MSCs. (**a**) Testes samples harvested 15 weeks after transplantation of hESCs (RH6) or hESC-MSCs under the testis capsule of nude mice. (**b**) H&E staining shows teratoma formation following transplantation of hESCs (RH6). (**c**) H&E staining shows normal testis following transplantation of hESC-MSCs. EC: Ectoderm; ME: Mesoderm; EN: Endoderm.

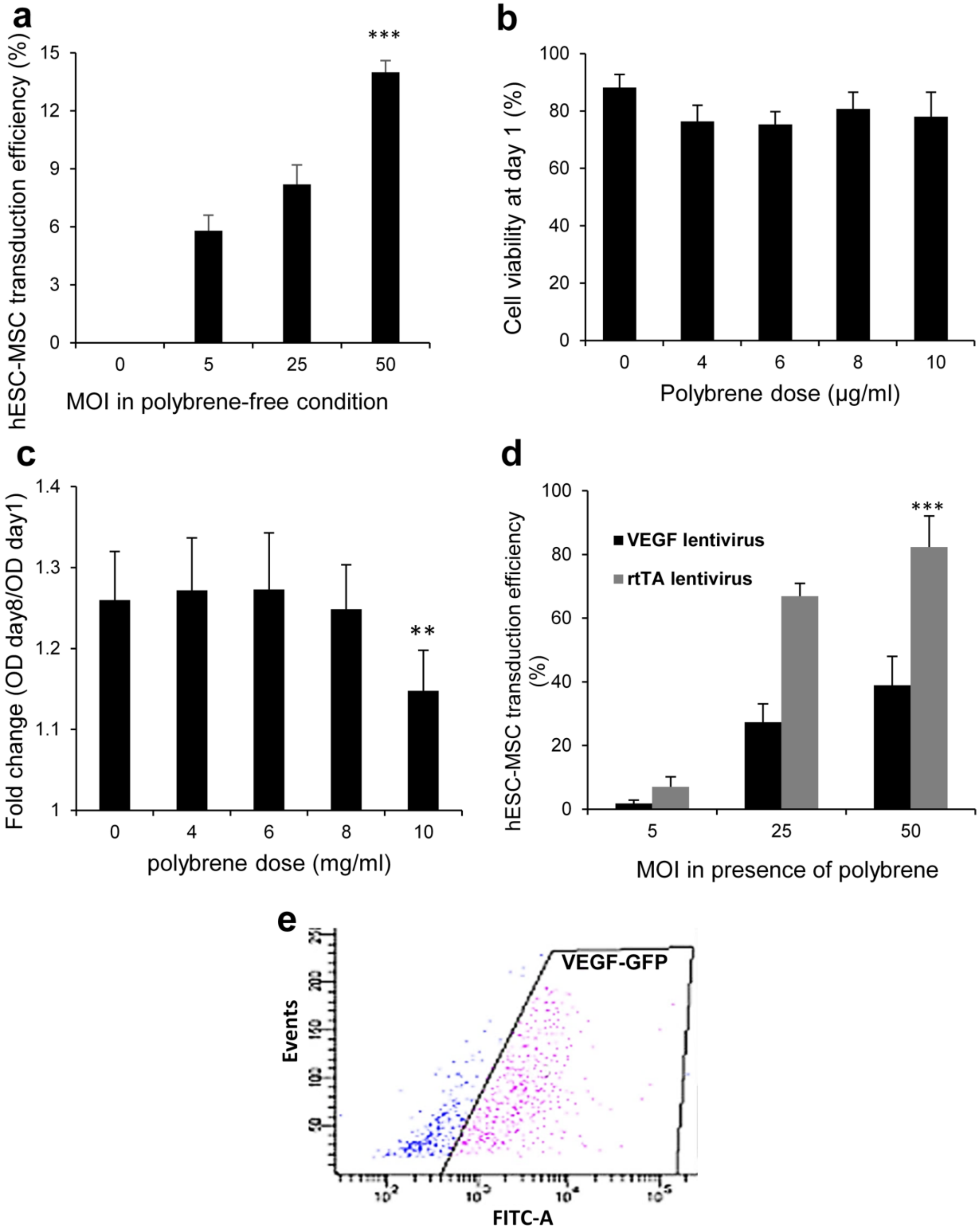

Supplementary Fig. S2. Optimization of lentiviral transduction conditions for hESC-MSCs. (**a**) hESC-MSC transduction efficiency with rtTA lentivirus under polybrene-free conditions (MOI=0 considered as mock). (**b**) Viability, evaluated by flow cytometry for PI, of hESC-MSCs

after 16 hours of exposure to different concentrations of polybrene (0-10 μg/ml). (**c**) Proliferation of polybrene-exposed hESC-MSCs analyzed by comparing the results of the MTS assay on day 8 to day 1. (**d**) Flow cytometry of GFP+ cells to determine hESC-MSCs transduction efficiency in the presence of polybrene. (**e**) FACS of hESC-MSCs transduced by VEGF lentivirus. Values represent the mean±SD, * $p<0.05$, **$p<0.01$, ***$p<0.005$. MOI: Multiplicity of infection, OD: Optical density.

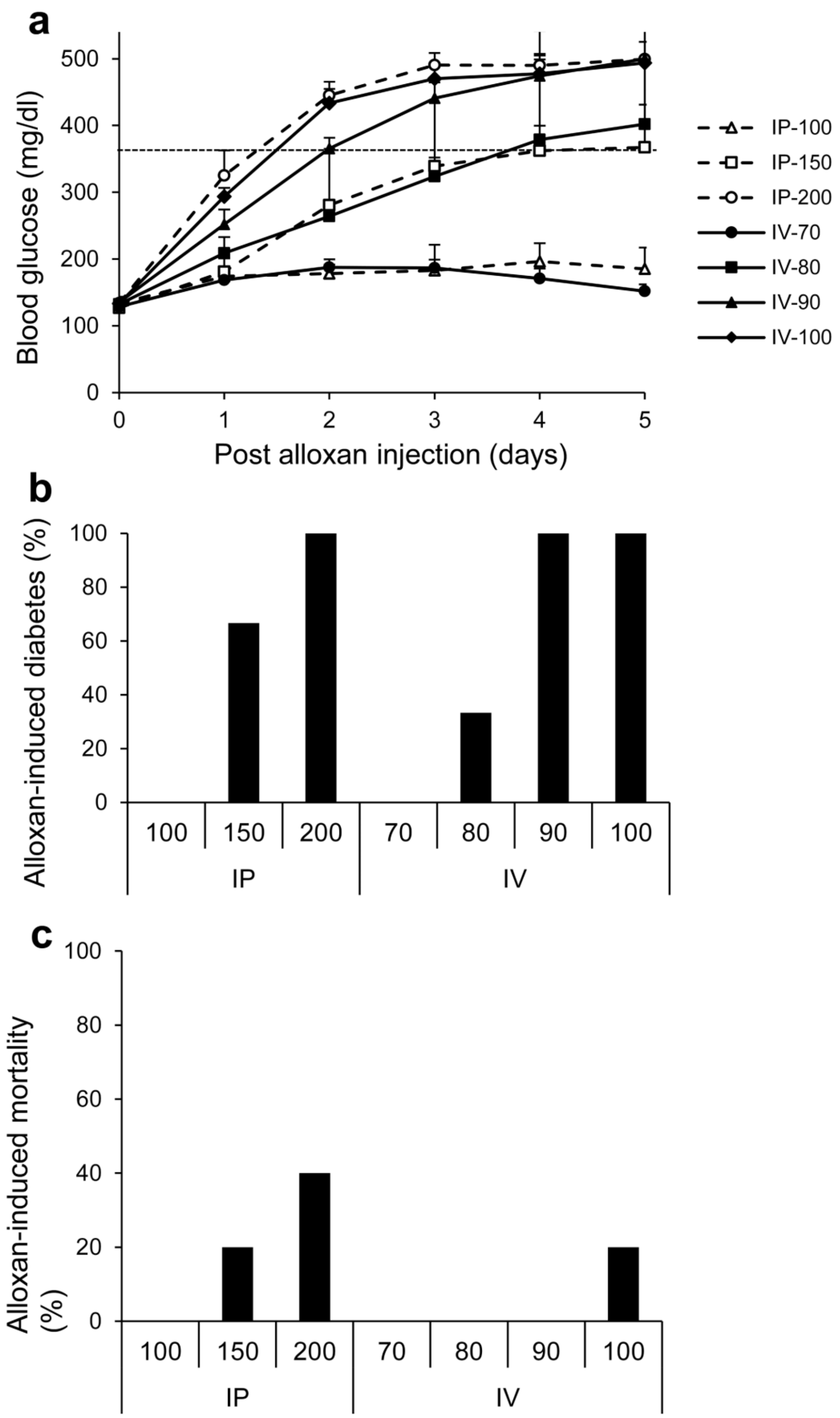
a
Blood glucose (mg/dl)
0
100
200
300
400
500
0
1
2
3
4
5
Post alloxan injection (days)
IP-100
IP-150
IP-200
IV-70
IV-80
IV-90
IV-100
b
Alloxan-induced diabetes (%)
0
20
40
60
80
100
100
150
200
70
80
90
100
IP
IV
c
Alloxan-induced mortality (%)
0
20
40
60
80
100
100
150
200
70
80
90
100
IP
IV

Supplementary Fig. S3. Optimization of diabetes induction by alloxan in B6NU mice. (**a**) Monitoring of non-fasting blood glucose concentrations after injection of different doses and administration routes of alloxan. (**b**) Diabetes induction rate after injection of different doses and administration routes of alloxan. (**c**) Mortality rate after injection of different doses and administration routes of alloxan. IP: Intra-peritoneal, IV: Intravenous.

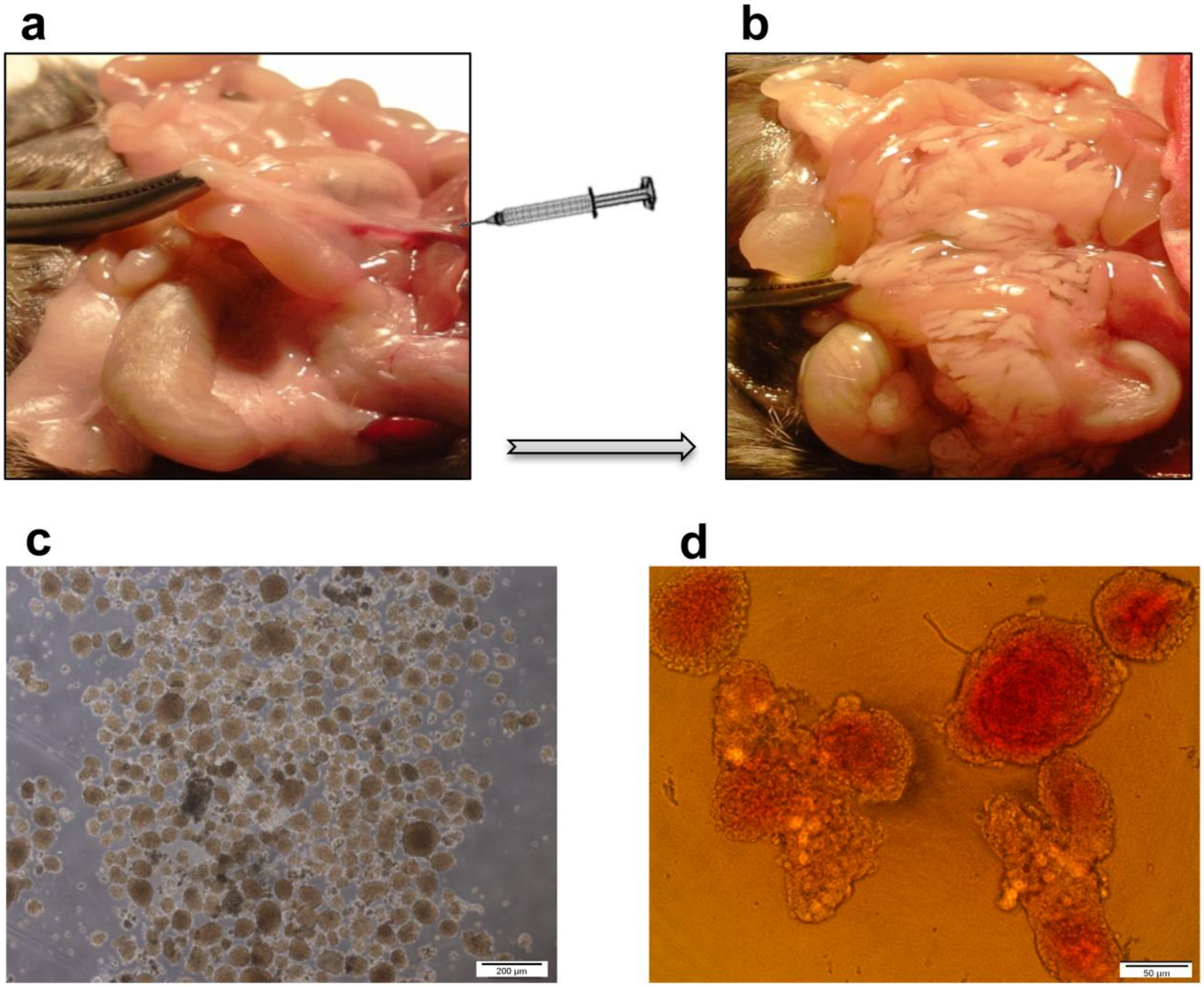

Supplementary Fig. S4. Optimization of mouse pancreatic islet isolation. (**a**) Injection of collagenase I solution into the pancreatic duct of C57BL/6 mice. (**b**) Congested pancreas after ductal injection of collagenase I solution. (**c**) Isolated pancreatic islets. (**d**) Purity of isolated pancreatic islets as evaluated by dithizone (DTZ) staining.

a

Step	Material	Concentration	Volume
1	Collagen I	15 mg/ml	30 µl
2	PBS	10X	5 µl
3	NaOH	0.4 N	5 µl
4	5 min incubation on ice		
5	Fibrinogen	15 mg/ml	45 µl
6	Islet +/- Cell		40 µl
7	Thrombin	50 U/ml	15 µl

b c

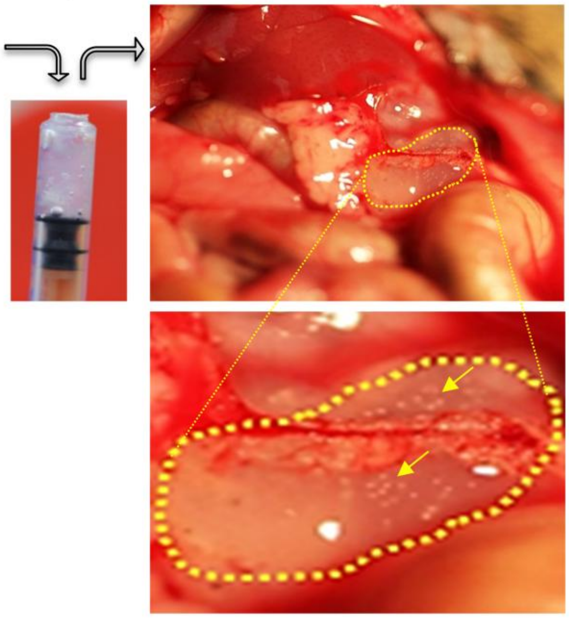

Supplementary Fig. S5. Transplantation of islet-containing hydrogel into the omental pouch of diabetic mice. (**a**) Steps for preparation of islet-containing collagen-fibrin hydrogel. (**b**) Transfer of islet-containing hydrogel via a cut-end insulin syringe. (**c**) Transplantation of islet-containing hydrogel into omental pouch, avoiding damage to the omental blood vessels.

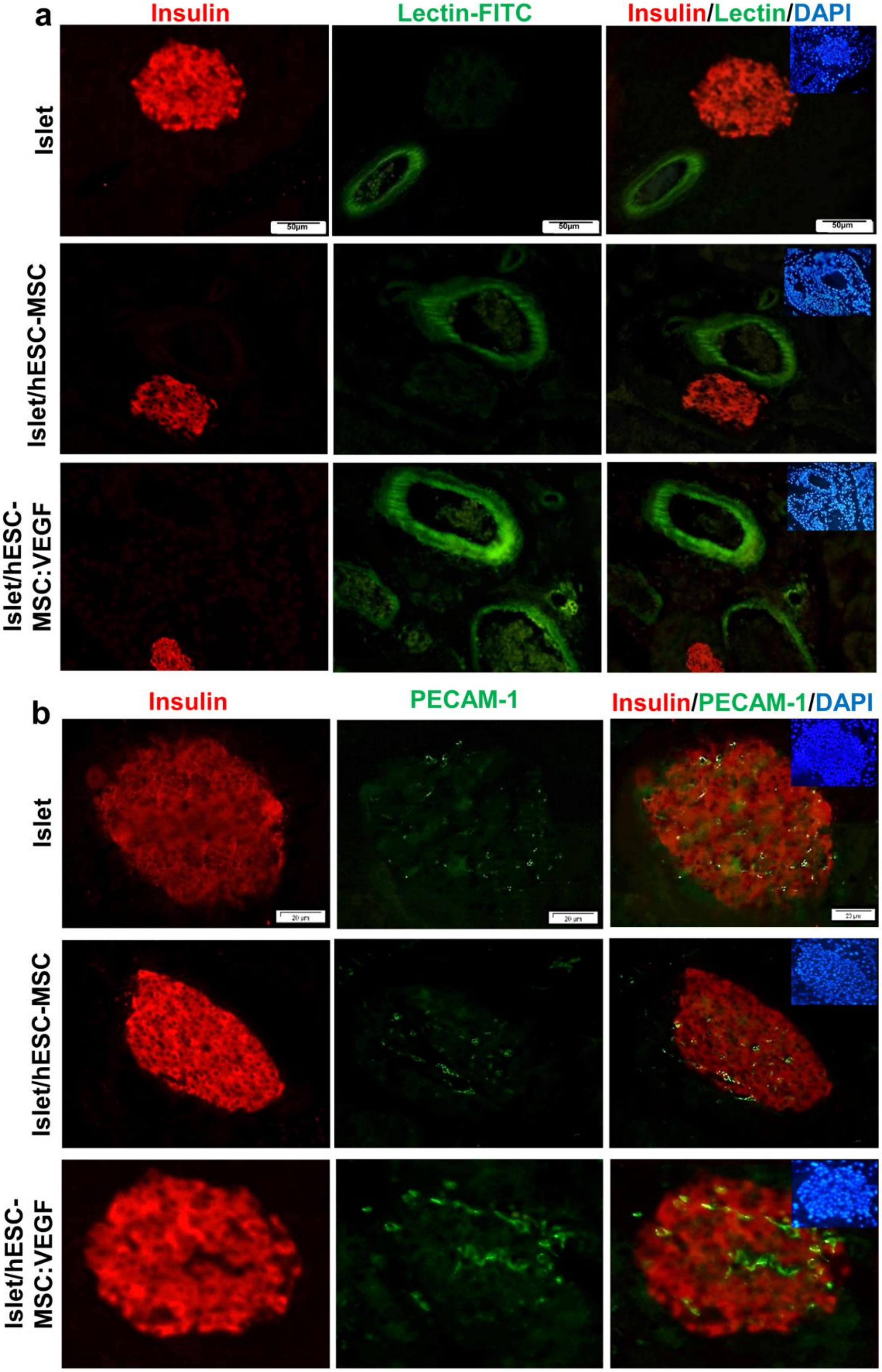
a
Insulin
Lectin-FITC
Insulin/Lectin/DAPI
Islet
Islet/hESC-MSC
Islet/hESC-MSC:VEGF
50μm
b
Insulin
PECAM-1
Insulin/PECAM-1/DAPI
Islet
Islet/hESC-MSC
Islet/hESC-MSC:VEGF
20 μm

Supplementary Fig. S6. Immunostaining for insulin and blood vessel markersin islet grafts at four weeks after transplantation. (**a**) Fluorescein isothiocyanate–conjugated tomato lectin (FITC-lectin) was injected into the tail vein. After processing the harvested graft, goat anti-mouse insulin primary antibody and donkey anti-goat alexafluor 594 secondary antibody were applied to the slides. DAPI: 4´,6-diamidino-2-phenylindole dihydrochloride. (**b**) Co-staining of the graft sections with insulin and PECAM-1 antibodies.
